# Mechanisms underlying dual effects of serotonin during development of *Helisoma trivolvis* (Mollusca)

**DOI:** 10.1186/1471-213X-14-14

**Published:** 2014-03-13

**Authors:** Konstantin Glebov, Elena E Voronezhskaya, Marina Yu Khabarova, Evgeny Ivashkin, Leonid P Nezlin, Evgeni G Ponimaskin

**Affiliations:** 1 Department of Neurology, University Hospital of Bonn, Bonn, Germany; 2 DFG-Research Center Molecular Physiology of the Brain (CMPB), Göttingen, Germany; 3 Institute of Developmental Biology RAS, Moscow, Russia; 4 Department of Experimental Neurocytology, Brain Research Branch, Scientific Center of Neurology, RAMS, Moscow, Russia; 5 Department of Cellular Neurophysiology, Carl-Neuberg-Str. 1, 30625 Hannover, Germany

**Keywords:** Serotonin receptors, *Helisoma trivolvis*, Development, Metamorphosis, Trochophore, Larval competence

## Abstract

**Background:**

Serotonin (5-HT) is well known as widely distributed modulator of developmental processes in both vertebrates and invertebrates. It is also the earliest neurotransmitter to appear during neuronal development. In aquatic invertebrates, which have larvae in their life cycle, 5-HT is involved in regulation of stages transition including larval metamorphosis and settlement. However, molecular and cellular mechanisms underlying developmental transition in aquatic invertebrate species are yet poorly understood. Earlier we demonstrated that in larvae of freshwater molluscs and marine polychaetes, endogenous 5-HT released from the neurons of the apical sensory organ (ASO) in response to external stimuli retarded larval development at premetamorphic stages, and accelerated it at metamorphic stages. Here we used a freshwater snail *Helisoma trivolvis* to study molecular mechanisms underlying these dual developmental effects of 5-HT.

**Results:**

Larval development of *H. trivolvis* includes transition from premetamorphic to metamorphic stages and shares the main features of metamorphosis with free-swimming aquatic larvae. Three types of 5-HT receptors (5-HT_1_-, 5-HT_4_- and 5-HT_7_-like) are functionally active at premetamorphic (trochophore, veliger) and metamorphic (veliconcha) stages, and expression patterns of these receptors and respective G proteins undergo coordinated changes during development. Stimulation of these receptors modulated cAMP-dependent regulation of cell divisions. Expression of 5-HT_4_- and 5-HT_7_-like receptors and their downstream G_s_ protein was down-regulated during the transition of pre- to metamorphic stage, while expression of 5-HT_1_ -like receptor and its downstream G_i_ protein was upregulated. In accordance with relative amount of these receptors, stimulation of 5-HTRs at premetamorphic stages induces developmental retardation, while their stimulation at metamorphic stages induces developmental acceleration.

**Conclusions:**

We present a novel molecular mechanism that underlies stage-specific changes in developmental tempo of *H. trivolvis* larvae in response to endogenous 5-HT produced by the neurons of the ASO. We suggest that consecutive changes in expression patterns of different receptors and their downstream partners in the course of larval development represent the molecular base of larval transition from premetamorphic (non-competent) to metamorphic (competent) state.

## Background

Serotonin (5-hydroxytryptamine, 5-HT) is well known not only as neurotransmitter modulating wide range of neurophysiological processes, but also as one of the earliest to appear and widely distributed modulator of development in both vertebrate and invertebrate animals [1-8]. The hypothesis that 5-HT plays a critical role as growth regulatory signal in the developing brain [3,9,10] was confirmed by subsequent studies [8,11]. In addition to its role in neuronal development, 5-HT is implicated in regulation of various developmental processes, i.e., meiosis reinitiating, cell divisions, morphogenetic movements, and left-right axis patterning [4-6,12-15].

The great majority of aquatic animals have a complex biphasic life cycle in which a free-swimming or encapsulated larval phase precedes juvenile and adult form. Larval development is a complex process controlled by various external and internal factors [16,17], and 5-HT has been shown to be involved in regulation of stages transition including larval metamorphosis and settlement [16]. For example, 5-HT mediated the induction of dauer larva formation in the nematode *Caenorhabditis elegans*[18,19], stimulated metamorphosis in hydroid *Eudendrium racemosum* in the presence of a natural cue [20], induced metamorphosis in scyphozoan and hydrozoan planulae [21,22], and induced larval settlement in barnacles [23] and molluscs [24,25]. In ascidian larvae, 5-HT signalling was suggested to trigger metamorphosis [26]. In all listed studies, 5-HT was suggested to act via larval nervous system and downstream 5-HT receptors. Thus, 5-HT receptors (5-HTRs) with pharmacological profiles similar to those of mammalian 5-HT_1_R and 5-HT_2_R were demonstrated to mediate rotational response to hypoxia in trochophore larvae of pulmonate snail *Helisoma trivolvis*[27-29], and 5-HT_1_R and 5-HT_7_R were cloned [30]. Two receptors with high and low affinities for serotonin mediated settlement in cyprid larvae of barnacle *Balanus amphitrite*[23] and G protein-coupled receptor homologues to 5-HT_1_R were cloned for this species [31]. Pharmacological analysis also suggested involvement of 5-HT_1_R in induction of metamorphosis of competent planula of hydroid *E. racemosum*[20]. However, information about 5-HT receptor subtypes and particularly about their signal transduction systems underlying effects of 5-HT during larval development and metamorphosis is scanty, and usually only one particular developmental stage (either early premetamorphic, or metamorphic) was examined, whereas changes during development were not traced.

It is known that presence of conspecific adults can influence larval development. Thus, water-born chemicals from conspecific adults delayed hatching in crustaceans [32,33] and accelerated time-to-hatching in the marine gastropod *Nucella lamellose*[34]. Previously we found that larvae of freshwater molluscs *L. stagnalis* and *H. trivolvis* and marine polychaete *Platynereis dumerilii* can regulate developmental tempo (the speed of transition from one stage to another) in response to a chemical signal emitted by starved conspecific adults. In our experiments, the signal retarded larval development at premetamorphic stages and accelerated it at metamorphic stages [35,36]. We also demonstrated that changes in the developmental rate in respond to the chemical signal were transmitted via 5-HT neurons of the apical sensory organ (ASO) and 5-HT released by these neurons was involved in developmental regulation [35]. However, molecular mechanisms responsible for stage-dependent dual effects of endogenous 5-HT remain unknown.

The ASO is a specialized and highly evolutionary conserved larval structure composed of sensory and motor neurons, which allow the larvae to adapt its behaviour to changing environment [37-40]. A characteristic feature of the ASO across larvae of diverse invertebrate phyla is a group of serotonergic cells [1,39,41-43]. In many aquatic invertebrates, these neurons are the first to differentiate and represent the central component of the larval nervous system [41-45]. The neurons of the ASO coordinate ciliary activity for swimming and feeding at early larval stages [27,29,46,47] and play a critical role in perceiving settlement-inducing cues leading to metamorphic transition in competent larvae [17,24,48,49]. In all this cases, 5-HT produced and released by apical neurons exerts its effects through activation of respective 5-HT receptors.

In the present study we used a freshwater gastropod *Helisoma trivolvis* to analyse the molecular mechanisms underlying the physiological effects of 5-HT during premetamorphic and metamorphic larval stages. We consider *H. trivolvis* as a suitable model to address this question by several reasons. Firstly, although the whole development of *H. trivolvis* until the stage of a juvenile snail occurs inside egg capsules, the embryos pass the same developmental stages as free-swimming larvae of trochophore animals: trochophore, veliger and veliconcha [35,47,50,51], and these stages can be easily determined according to the set of morphological and morphometric characters (larval ciliary bands and apical neurons, as well as the degree of shell, tentacles and foot development, eye pigmentation, and type of locomotion). Secondly, normal development of all embryos within one egg mass is highly synchronous, and the length of the embryos at subsequent developmental stages precisely correlates with the above listed morphological and behavioural characters [35], thus measuring the length can be used as a valid indicator of stage progression. Thirdly, two 5-HT receptors in *H. trivolvis* (5-HT_1_R and 5-HT_7_R) have been recently cloned [30], thus making possible to monitor the relative expression of these receptors at different developmental stages.

Using pharmacological and molecular approaches we demonstrate functional involvement of at least three types of 5-HTRs in the regulation of *H. trivolvis* developmental tempo. We found that activation of 5-HT_4_-like receptor (5-HT_4_R) and 5-HT_7_-like receptor (5-HT_7_R) induced developmental retardation, while activation of 5-HT_1_-like receptor (5-HT_1_R) induced developmental acceleration. This work has appeared previously in abstract form [52]. Expression patterns of 5-HT_1_R, 5-HT_7_R and corresponding G_i_ and G_s_ proteins changed in a certain manner at premetamorphic and metamorphic developmental stages. Based on our experimental results we suggest a molecular mechanism by which 5-HT regulates larval development in alternative ways depending on the developmental stage (premetamorphic or metamorphic) the larva passes.

## Methods

### Animals and identification of developmental stages

Egg masses were collected from *H. trivolvis* inbred colony maintained at the Institute of Developmental Biology, Moscow. Ten to twelve mature snails were raised in plastic tanks with a 16/8 h light/dark cycle at 23-25°C and fed on lettuce. Freshly laid egg masses were collected daily and transferred to Petri dishes (Falcon) containing boiled filtered (0.2 μM Millipore) pond water. Each adult snail produces daily 1–2 transparent egg masses containing 10–30 eggs each.

Embryonic development was staged on the basis of specific transient larval characters such as dorsolateral ciliary bands (the remnants of the prototrochal ciliary band), pedal ciliary band and apical 5-HT-like immunoreactive neurons (remnants of the ASO), as well as developing adult morphological and behavioural features: the degree of shell, tentacles and foot development, eye pigmentation, and type of locomotion [50,51]. Stage 19 corresponds to early premetamorphic trochophore (20% of normal embryonic development or n.e.d.), stage 23 - middle veliger (45% n.e.d.), stage 25 – veliconcha (65% n.e.d.), stage 27 - the last larval stage (85% n.e.d.) and stage 28 - early adult-like form (90% n.e.d.).

Since the increase in size of the embryos is in correlation with successive developmental stages [35,50], we checked what size corresponds to particular stages determined according to the set of morphological, morphometric and behavioural characters (Table 1). In subsequent experiments, the length of embryos was used as valid indicator of stage progression. Egg masses at appropriate developmental stages were selected for experiments. Each egg contains a single embryo and development of all embryos in each egg mass is synchronous under standard conditions. However, embryos within a single egg mass develop independently and their responses to external stimuli can vary, thus each embryo can be treated as an independent sample.

**Table 1:** **Normal development of *H. trivolvis***

**Stage name**		**Stage after Meshcheriakov**	**Stage,%**	**Size, mm**
				**(min - max)**
Trochophore	Early	19	20	0.16 – 0.19
	Middle	20	25	0.20 – 0.24
	Late	21	30	0.25 – 0.31
Veliger	Early	22	35	0.32 – 0.40
	Middle	23	45	0.41 – 0.47
	Late “hippo”	24	55	0.48 – 0.56
Metamorphic	Veliconcha	25	65	0.57 – 0.61
		26	75	0.62 – 0.66
		27	85	0.67 – 0.72
Adult-like		28	90	0.73 – 0.78
		29	100	0.74 – 0.79

Interrelations between developmental stages after Mescheriakov [51], timing of embryogenesis in%, and the length of specimens in mm are shown. Five hundred specimens were measured.

### Pharmacological treatments

The following drugs were used (Tocris, Bristol, UK, unless other specified): α-methylserotonin maleate, 5-carboxyamido-tryptamine maleate (5-CT), 2,5-dimethoxy-4-iodoamphetamine (DOI), forskolin (Sigma), GR113808, trimethyl 5-HT iodide (5-HTQ), (±)-8-Hydroxy-2-dipropylaminotetralin hydrobromide (8-OH-DPAT), methiothepin maleate, pimozide, RS67333, RS67506, SB269970, (S)-WAY100135 dihydrochloride, dibutyryl-cAMP sodium salt (db-cAMP), MDL 12330A hydrochloride, H 89 dihydrochloride. All solutions were prepared as 10 mM stocks and stored at −20°C. RS67333 and GR113808 were prepared as 10 mM solutions in DMSO. Equal amount of DMSO was added to the controls, and was shown to have no effect on the development. Receptor antagonists were applied 10 min before the agonists were added. For pharmacological experiments, egg masses were transferred into 30 mm dishes containing 2 ml of solution in appropriate concentration, the dishes were kept in a dark humid chamber at 25°C and solutions were changed daily.

In preliminary experiments, solutions of the agonists (RS67333, 5CT, 8-OH-DPAT) were changed after 12, 24, 48, and 72 hours. No significant differences were observed in the effects of the drugs when changed after 12 to 48 hours. When the solutions were changed after 72 hours, the effects became weaker. Thus, 24 hours were selected for all subsequent experiments. Efficacy of the drugs tested was determined according to the following criteria: the effects had to be significant, concentration dependent and manifest themselves at micromolar concentrations. In this case, we considered the drug to penetrate the egg casing and reach specific targets.

Egg masses containing 15–20 eggs were selected for each experiment. At least three sets of experiments were made for each treatment utilizing a blind experimental method to ensure impartiality. For investigation of cell divisions, egg masses were incubated in solutions of respective agonists during 10 h at 25°C then fixed and proceeds for immunocytochemistry.

### Immunocytochemistry

Embryos at the appropriate stage were gently removed from the egg capsules, washed from the egg fluid in water and then fixed in 4% paraformaldehyde in 0.01 M phosphate buffer (Sigma, PB, pH 7.4) for 4 h at 10°C and further processed as described earlier [35]. To visualize serotonergic neurons and ciliary bands, the specimens were incubated in a mixture of anti-5-HT (ImmunoStar, Hudson, USA, #20080, polyclonal, rabbit, dilution 1:3000) and anti-acetylated α-tubulin antibody (Sigma-Aldrich, Munich, Germany, T-6793, monoclonal, mouse, dilution 1:4000) in PB with 10% normal goat serum, 0.25% bovine serum albumin, 5% Triton X-100 (TX), and 0.03% sodium azide (PB-TX) for 72 h at 10°C. The specimens were washed in PB three times for 20 min, incubated in a mixture of goat-anti-rabbit Alexa 488-conjugated IgG and goat-anti-mouse Alexa 633-conjugated IgG (Molecular Probes, USA), both diluted 1:800 in PB-TX, for 12 h at 10°C. Finally, the samples were washed twice in PB, immersed in 70% glycerol in PB and mounted on glass slides. Morphological data presented in this study are based upon examination of at least fifteen embryos from at least three different egg masses for each particular stage described.

To visualize cell divisions, fixed embryos were washed in PB and incubated with the antibody against phosphorylated Ser28 histone H3 (Sigma-Aldrich, H9908) diluted 1:1000 in PB-TX for 72 h at 10°C. The specimens were washed three times in PB and incubated in goat-anti-rat IgG conjugated with Alexa 546 (1:800 dilution in PB-TX) for 12 h at 10°C. Samples were incubated for 10 min in DAPI (2.5 μg/ml in PB), washed two times in PB, immersed in 70% glycerol in PB and mounted on glass slides.

Stained embryos were examined as whole mounts under TCS SP5 laser-scanning microscope (Leica Microsystems, Wetzlar, Germany) with appropriate configuration settings. To determine the exact position of cells and ciliary structures, fluorescence images were combined with transmission light images. The number of nuclei stained with the antibody was counted from the maximal intensity projections using ImageJ (NIH, Bethesda, USA).

For illustrations, series of optical sections (0.7 μM step size, from the upper till the lower surfaces of the embryo) were projected into single images with greater focal depth using LAS-AF software (Leica Microsystems) and exported as TIFF images. All images were linearly adjusted for contrast and brightness and assembled into plates using Photoshop CS5 (Adobe, San Jose, USA).

### Measurements and statistics

The length of embryos was measured twice a day as described elsewhere [35]. Only the embryos being in the strict side view position were measured. Results were expressed as mean ± standard deviation (s.d.) or standard error of the mean (s.e.m.) and normalized to the respective control. The linear fitting of larval length and the slope coefficients were calculated using MS Excel 2007. To double check identification of developmental stages, some embryos were processed for immunocytochemical visualization of 5-HT neurons and ciliary structures. The significance of differences among groups was evaluated using one-way ANOVA, differences were considered significant at *p <*0.05.

### Quantitative real-time RT-PCR

*H. trivolvis* embryos were aspirated from eggs with a glass micropipette and immediately frozen in liquid nitrogen. Ten embryos from three different egg masses were used for each developmental stage at one trial. Three independent trials were performed for each stage. To release total RNA, cells were destroyed by repeated thawing and freezing in liquid nitrogen. Crushed embryos were incubated with RNase-free DNase I (2 U/sample, Life technologies) to remove genomic DNA contamination. After the inactivation of DNase I by heating, cDNA was synthesized by the Superscript II reverse transcription kit with random hexamer primers (Life technologies). Quantitative real-time PCR (ABI PRISM 7000, Life technologies, Bethesda, USA) was performed by using gene-specific primers and SYBR Green Master Mix kit (Life technologies). The following primers were used to amplify cDNA fragments of 5-HT_1_ receptor gene (GenBank accession no. AY395746): sense (5′-TCA ACA GCC TAC TGA ACC CTA TCA-3′) and antisense (5′-TCG GTG GCC CCG TCT ATA-3′). The following primers were used for the 5-HT_7_ receptor (GenBank accession no. AY395747): sense (5′-CCT CTG CGG TTA TGT CAA-3′) and antisense (5′-ACC GGA ACA GGA GAA TCT CTT TAA A-3′). For quantitative analysis, β-actin (GenBank accession no. AF435729) was analyzed in parallel by using sense (5′-CTT CAA CAC ACC AGC TAT GTA CGT T-3′) and antisense (5′-TTA CAC CGT CAC CAG AGT CCA T-3′) primers. The analysis was performed using ΔΔCt method according to the procedure described on: http://www3.appliedbiosystems.com/cms/groups/mcb_support/documents/generaldocuments/cms_042115.pdf.

### GTPgamma-Eu binding assay

*H. trivolvis* embryos at developmental stage 23 were removed from the egg capsules, washed with PB, resuspended in extraction buffer (10 mM Tris–HCl, pH 7,4; 1 mM EDTA; 150 mM NaCl; 1 mM DTT) and homogenized. The homogenate was centrifuged at 5.000 g for 5 min at +4°C, and resulting supernatant was centrifuged at 14.000 g for 50 min. The membrane pellets were resuspended in TE buffer (pH 7.4) and stored at −84°C. For the GTPγ-EU assay membranes were resuspended in assay buffer (50 mM Tris–HCl, pH 7.4; 2 mM EDTA; 100 mM NaCl; 3 mM MgCl_2_). 5-HT-promoted binding of GTPγ-Eu to defined G-proteins was performed using Perkin Elmer DELFIA GTP-Eu Binding Kit (Cat. No. AD0167) according to the manufacturer instructions and as described previously [53]. Briefly, 4 μl of membranes prepared from the embryos (10 μg per probe) were resuspended in 94 μl of assay buffer (50 mMTris-HCl (pH 7.4) containing 2 mM EDTA, 100 mMNaCl, 3 mM MgCl_2_,1 μM GDP). Serotonin at final concentration of 10 μM was added and membranes were incubated at RT for 10 min. FMRFamide at final concentration of 10 μM was used as positive control of G_q_ activation. After adding 1 μl GTP-Eu (PerkinElmer), samples were incubated for 1.5 h at RT. The reaction was terminated by adding 200 μl of termination buffer (50 mM Tris–HCl, pH 7.5; 20 mM MgCl_2_; 150 mM NaCl; 0,5% NP-40; 200 μg/ml aprotinin; 100 μM GDP; 100 μM GTP) for 15 min on ice. The samples were incubated with 10 μl of antibodies against G_s_ and G_q_, (sc823, sc392, respectively, all Santa Cruz, Dallas, USA) at RT followed by incubation with 75 μl of a Sepharose-Protein G conjugate (Sigma). Antibodies against anti-acetylated α-tubulin (T-6793, Sigma-Aldrich, Munich, Germany) were used as a control for the unspecific binding during immunoprecipitation. Immunoprecipitates were washed three times in termination buffer, heated at 99°C for 3 min in 0.2 ml of 0.5% SDS, centrifuged at 13.000 g for 5 min and the fluorescence intensity of supernatant was measured at 615 nm in multi-fluorescence plate reader Mithras LB680 (Berthold).

### Western blot

Expression of the 5-HT_1_R, α-tubulin, G_s_-, G_q_- and G_i_-protein was studied using membrane preparations of *H. trivolvis* at stages 19, 23 and 25. Embryos were removed from the eggs, washed with PB, resuspended in extraction buffer (10 mM Tris–HCl, pH 7.4, 1 mM EDTA, 150 mM NaCl, 1 mM DTT) and homogenized by a rotating pestle at 2000 r.p.m. 1000 embryos at stage 19, 600 at stage 23, and 400 at stage 25 were used for membrane preparation used for one trial. Tissue homogenate was centrifuged at 5.000 g for 5 min at +4°C, and the resulting supernatant was centrifuged at 14.000 g for 50 minutes. The membrane pellets were resuspended in TE buffer (pH 7.4) and stored at −84°C. Preparations were analysed by SDS-PAGE on 12% acrylamide gel and visualized by filmless chemiluminescence analysis (PeqLab). Primary antibodies against α-tubulin (Sigma-Aldrich, T-6793), 5-HT_1A_R (sc-10801), 5-HT_4_R (sc-28959 and AS9459, [26]), 5-HT_7_R (sc-28963), G_s_ and G_i_ proteins (sc-823, sc-12798, respectively, all Santa Cruz, Dallas, USA) were used. Preincubation of the antibody with corresponding blocking peptide (sc-823P, sc-392P, sc-12798P, all Santa Cruz) was used to verify the specificity of response and resulted in elimination of specific bands. Five trials were performed for each developmental stage. For quantitative analysis, the Scan Pack 2.0 software (Biometra, Goettingen, Germany) was used.

## Results

### Developmental analysis of *Helisoma trivolvis*

**Figure 1:**
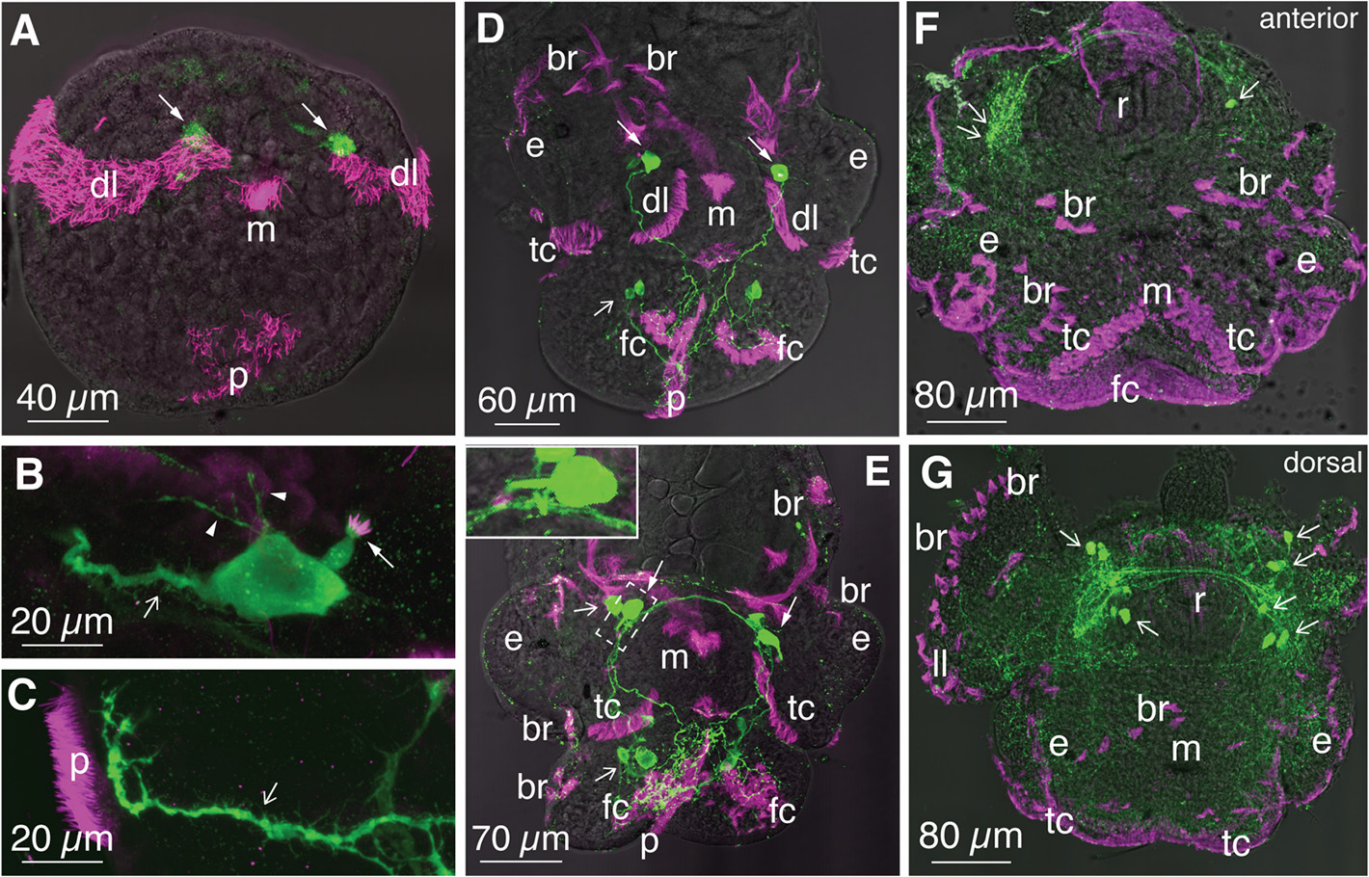
**Development of ciliary structures and serotonergic system in *H. trivolvis.*** Anti-α-tubulin (magenta) and anti-5-HT (green) immunostainings were imaged in a laser confocal microscope. In **A** and **D-G**, confocal images were combined with transmission light images. **(A-C)** Early trochophore (stage 19). **(A)** Two apical neurons (arrows), dorsolateral (dl) and pedal (p) ciliary bands are present. **(B)** Higher magnification of the apical neuron with dendritic knob, which bears short cilia (arrow), thin back branches (arrowheads), and primary neurite (open arrow). **(C)** Primary neurite (open arrow) with numerous thin branches underneath the pedal band of cilia (p). **(D)** Middle veliger in the beginning of metamorphosis (stage 23). Dorsolateral and pedal ciliary bands are complemented by foot ciliary fields (fc), tentacle ciliary fields (tc) and numerous body wall ciliary brushes (br). Two apical 5-HT neurons (arrows) and 5-HT positive cells within cerebral and pedal ganglia (open arrows) are present. **(E)** veliconcha in the middle of metamorphosis (stage 25). Dorsolateral ciliary bands are not visible, whereas all other ciliary structures are present. *Insert*, high magnification of the apical 5-HT neuron with ciliated dendritic knob. **(F, G)***H. trivolvis* at the end of metamorphosis (stage 27), anterior **(F)** and dorsal **(G)** view. Serotonin-immunostaining is present in neurons of central ganglia (open arrows) and their peripheral processes only. Foot and tentacle ciliary fields (fc, tc) and numerous body wall ciliary brushes (br) are present. Note that 5-HT-immunopositive apical neurons, cilia on their dendritic knobs as well as dorsolateral and pedal ciliary bands are not visible. ll – lung lobe, m – mouth, e – eye, r – radular sack.

Although the general development of freshwater gastropods has been previously described [47,50,51,54,55], formation of transitory larval characters in *H. trivolvis* during the whole development has not been addressed in details. In *H. trivolvis*, initial cleavage resulted in formation of a ciliated trochophore larva (stage 19), which continually rotated in the egg fluid using dorsolateral and pedal ciliary bands. At this stage, two 5-HT-positive neurons were located dorsally to the mouth at the base of each dorsolateral ciliary band (Figure 1A). Each neuron had a dendritic knob that bore short cilia (Figure 1B). Thin branches emanated from the neuronal body and ran below the cilia of the dorsolateral band (Figure 1B). A thick primary neurite projected to the larval foot where numerous thin branches ramified underneath the pedal ciliary band (Figure 1C).

At stage 23, the larva became a middle veliger. Slow basal rotation started to alternate with transient periods of acceleration and occasional stops. Tentacular ciliary bands, symmetrical foot ciliary fields and numerous ciliary brushes added to dorsolateral and pedal ciliary bands (Figure 1D). In addition to apical serotonergic neurons, one 5-HT immunoreactive neuron in each cerebral ganglion and two neurons in each pedal ganglion were present at this stage (Figure 1D).

At stage 25, the larvae entered the metamorphosis. At the region of developing head, only tentacular ciliary bands and ciliary brushes could be observed, while dorsolateral ciliary bands were no more detected (Figure 1E). Additional ciliary brushes appeared on the lateral sides of the foot; symmetrical foot ciliary fields enlarged to merge, while the pedal ciliary band was still visible in the medial ventral area of the foot. Among 5-HT-immunopositive structures, commissures and connectives of the ganglionic ring as well as projections from the ganglionic neurons to the foot, tentacles and body wall became visible (Figure 1E). At this stage, up to three 5-HT positive neurons could be recognized in each pedal ganglion. Apical 5-HT neurons maintained their positions dorsolateral to the mouth and outside the rudiments of the cerebral ganglia. Ciliated dendritic knobs of these cells (Figure 1E, insert) as well as their projections and ramifications underneath the pedal ciliary bands were clearly visible.

During stages 26 and 27, metamorphosis progressed and by the end of stage 27 swimming movements ceased, the embryo attached its fully developed foot to the inner surface of the egg capsule and started to creep along it. By the end of stage 27, the enlarged tentacular ciliary band and numerous body wall ciliary brushes were present at the head region. Enlarged symmetrical ciliary fields on the foot merged and formed a unified ventral ciliated surface of the foot, thus the pedal ciliary band could no longer be recognized (Figure 1F, G). 5-HT-immunopositive neurons of the cerebral ganglia and their peripheral projections to the body wall, tentacles and foot were visible, while neither the 5-HT immunopositive cell bodies of the apical neurons, nor their ciliated knobs could be detected (Figure 1F, G).

Thus, according to the presence of transitory larval characters such as dorsolateral and pedal ciliary bands and 5-HT-immunoreactive apical neurons, we consider developing *H. trivolvis* to be premetamorphic larvae at stages 19–23, metamorphic larvae at stages 25–27 and postmetamorphic adult-like forms at stage 28 and later. The duration of development from the trochophore (stage 19) till the adult-like postmetamorphic snail (stage 28) varied depending on the temperature. At 25 ± 0.5°C it took 88 ± 7.2 h (n = 289), at 23 ± 0.5°C 108 ± 11.6 h (n = 194) and at 21 ± 0.5°C 162 ± 15.5 h (n = 90). The temperature 25 ± 0.5°C was selected for all further experiments because at this temperature the development of embryos was the most synchronous.

### Larval development is bidirectionally regulated by activation of different 5-HT receptors

**Figure 2:**
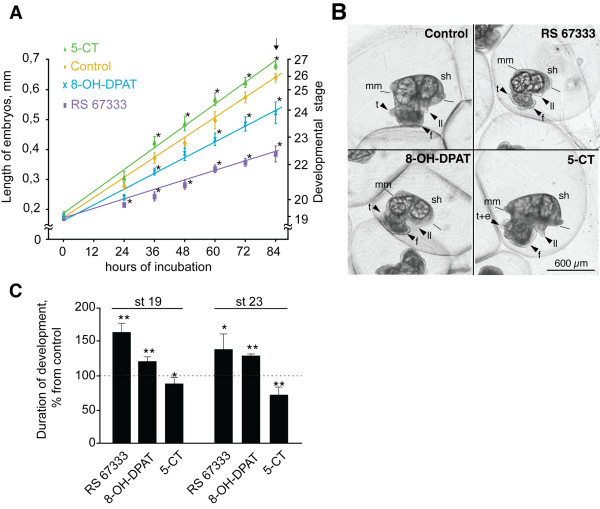
**Effects of different agonists of 5-HT receptors on embryonic development of *H. trivolvis. *(A)** The length of embryos during the development under control conditions (control, n = 80) and after incubation with 5-HT_4_R agonist RS67333 (2.5 μM, n = 80), 5-HT_7_R agonist 8-OH-DPAT (5 μM, n = 80) and 5-HT_1_R agonist 5-CT (1 μM, n = 80). Development is significantly retarded after RS67333 and 8-OH-DPAT, and accelerated after 5-CT treatment. Linear fitting slope coefficient (k) is 0.068 for control (R^2^ = 0.99), 0.031 for RS67333 (R^2^ = 0.98), 0.052 for 8-OH-DPAT (R^2^ = 0.99), and 0.075 for 5-CT (R^2^ = 0.99). **(B)** Overview of representative larvae after 84 h of incubation in the above mentioned drugs, equal magnification. The size of embryos strongly corresponds to certain developmental stages. At the time when the control animal is in the end of metamorphosis and represents a miniature snail, the embryos incubated with RS67333 and 8-OH-DPAT are still at the premetamorphic stage. On the contrary, the embryo incubated with 5-CT reaches the postmetamorphic adult-like stage and is bigger than the control. (See also supplementary materials Movies 1 to 3). f – foot, ll – lung lobe, mm – mantle margin, t – tentacle, e – eye, sh – shell. **(C)** Incubation with RS67333 (2.5 μM) and 8-OH-DPAT (5 μM) results in significant increase of both total duration of development and duration of metamorphosis. Retardation effect of RS67333 is more pronounced, when treatment starts at stage 19. Incubation with 5-CT (1 μM) reduces developmental time and duration of metamorphosis with the strongest effect at stage 23 (n = 60 for each group). Data are shown as mean ± s.d. (*, *p <*0.05; **, *p <*0.01).

To study the roles of 5-HTRs in larval development of *H. trivolvis*, we used the set of specific agonists. Specimens were continuously incubated in drug-containing solutions starting from the trochophore stage until the end of metamorphosis (stages 19 and 28, respectively). Agonists of mammalian 5-HT_2_R (α-methylserotonin and DOI) and 5-HT_3_R (5-HTQ) in concentrations 1 μM, 5 μM and 10 μM had no effect on embryonic development (n = 40 for each concentration for each drug tested). Incubation of larvae with RS67333 (agonist of the mammalian 5-HT_4_R) resulted in pronounced developmental retardation, which was expressed as elongation of each developmental stage and corresponding decrease in larval length compared to the controls at each observation point. This retardation started after 24 h of incubation and progressed by 84 h of incubation (Figure 2A, B). Significant retardation was also obtained after chronic treatment of embryos with 8-OH-DPAT (agonist of 5-HT_7_R and partial agonist of 5-HT_1_R; Figure 2A, B).

In contrast, prolonged incubation with 5-CT (agonist of 5-HT_1_R and partial agonist of 5-HT_7_R) accelerated larval development, so the experimental embryos outgrew the controls. Thus, by 72 h of incubation with 5-CT larval length was equal to that of the controls corresponding to 84 h of incubation (Figure 2A, B). The 5-CT-mediated acceleration of developmental rate started after 36 h of incubation and was persistent during the whole development (Figure 2A).

As a consequence of these receptor-mediated changes in the developmental rate, after 84 h of incubation (when control animals reached the middle of metamorphosis) the animals treated with RS67333 or 8-OH-DPAT were at premetamorphic stages only, while the animals treated with 5-CT already reached the end of metamorphosis (Figure 2B). To compare the speed of development, the linear fitting was applied to the measurements of embryonic lengths and the slope was calculated (Figure 2A). This analysis provided slope coefficients k_5-CT_ = 7.5×10^−2^ (R^2^ = 0.992) > k_control_ = 6.8×10^−2^ (R^2^ = 0.994) > k_8-OH-DPAT_ = 5.2×10^−2^ (R^2^ = 0.988) > k_RS67333_ = 3.1×10^−2^ (R^2^ = 0.976), where the higher value of the slope indicates the faster development.

Analysis of the specific set of morphological characters confirmed that differences in larval length correspond to different developmental stages. When control animals reached the middle of metamorphosis, the larvae exposed to RS67333 and 8-OH-DPAT (84 h of incubation) reached only the premetamorphic veliger stages. The control animals reached the stage 26: the shell covered the visceral mass; the foot, head tentacles and lung lobe developed, the eyes (though not yet pigmented) appeared at the base of the tentacles. The embryo attached the foot to the inner surface of the egg capsule and started to creep along it (gliding locomotion), though sometimes detached and continued rotational swimming. Additional movie files show this in more detail [see Additional file 1 and Additional file 2].

At the same time, the larvae exposed to RS67333 reached only the premetamorphic stage of early veliger (stage 22–23). They had a small foot, developing shell covered only the posterior half of the visceral mass, the tentacle rudiments and eyes just appeared. The larvae treated with 8-OH-DPAT also demonstrated developmental retardation, though not that profound (stage 24). They had an elongated foot distinctly separated from the head region, and the shell, which covered two-thirds of the visceral mass. In addition, the specimens treated with RS67333 and 8-OH-DPAT expressed only rotational swimming, which is characteristic for premetamorphic larvae (Figure 2B). For more detail see an additional movie file [see Additional file 3].

On the contrary, the animals treated with 5-CT were at the end of metamorphosis (stage 27) and looked like miniature adult snails (Figure 2B): the shell covered the whole visceral mass, and animals could withdraw their bodies into the shell, the eyes at the base of the head tentacles were pigmented and the lung lobe was elongated. These specimens showed no rotational swimming and crept along the inner surface of the egg capsule using the fully developed foot (Figure 2B). For more detail see an additional movie file [see Additional file 4]. No asynchrony in morphogenesis or disproportionality in growth of either inner (heart, larval liver, lung cavity, radular pouch) or outer organs (shell, lung lobe, foot, tentacles, eyes) was observed in all treated animals.

### Functional effects of 5-HTRs activation depend on the developmental stage

To test whether the effects of 5-HTRs activation depend upon developmental stages of larvae, we incubated the specimens with specific receptor agonists starting either from the early premetamorphic trochophore stage 19 or from the middle veliger stage, just before metamorphosis (stage 23) and finishing the treatment when metamorphosis was complete (stage 28; Figure 2C). For each developmental stage, a separate experiment with a control and a batch exposed to the respective agonist was performed. When treatment with the 5-HT_4_R agonist RS67333 started at the stage 19, it resulted in significantly prolonged duration of development from the stage 19 until the stage 28 as compared to the controls (167 ± 14.3%; n = 60, *p <*0.05; Figure 2C). The effect was less pronounced when treatment started from the stage 23 (140 ± 5.6%; n = 40, *p <*0.05, Figure 2C). Incubation with 8-OH-DPAT also resulted in prolongation of development until the stage 28, although the effect was weaker and stage-independent (123 ± 19.1% at stage 19; and 135 ± 5.6% at stage 23; n = 60, *p <*0.05; Figure 2C). The effect of incubation with 5-CT was opposite - the time of development from stage 19 until stage 28 was significantly shorter as compared to the controls. The effect of 5-CT was more pronounced, when treatment started at the stage 23 than at the stage 19 (90 ± 9% at the stage 19; and 72 ± 11.9% at the stage 23; n = 60, *p <*0.05; Figure 2C).

### Pharmacological analysis of 5-HTRs involved in developmental retardation and acceleration

**Figure 3:**
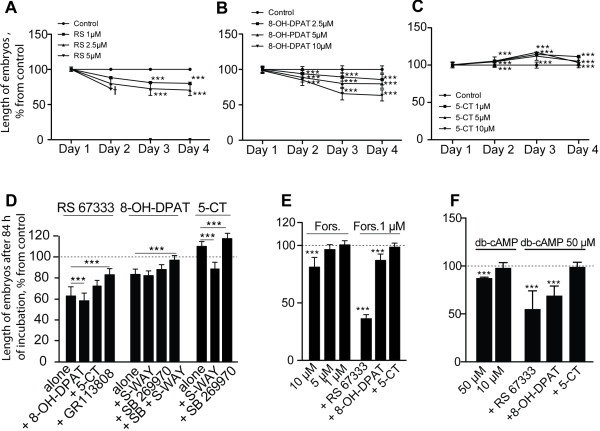
**Pharmacological analysis of 5-HT receptors involved in retardation and acceleration of *H. trivolvis *development. (A-C)**. Development tempo expressed as changes in the length of embryos is modulated upon treatment with different 5-HTR agonists in a dose-dependent manner. Treatment with RS67333 **(A)** and 8-OH-DPAT **(B)** results in retardation, while treatment with 5-CT **(C)** – in acceleration effects. **(D)** Effect of combined application of agonists and antagonists on the embryos length after 84 h of incubation. Data were normalized to the control and shown as mean ± s.d. (***, *p <*0.001). **(E)** Effect of forskolin treatment on the embryos length after 84 h of incubation. Normalized values represent means ± s.d.; (***, *p <*0.001). **(F)** Effect of db-cAMP treatment on the embryos length after 84 h of incubation. Normalized values represent means ± s.d.; (***, *p <*0.001).

We next analysed the dose-dependence of different agonists on the *H. trivolvis* developmental rate. As shown in Figure 3A, application of RS67333 at 5 μM concentration completely blocked development and caused 100% mortality after 84 h of incubation (n = 60). Application of 2.5 μM and 1 μM of RS67333 resulted in progressive retardation being more pronounced for the higher concentration (n = 60, *p <*0.001; Figure 3A). Treatment with different concentrations of 8-OH-DPAT resulted in similar effects: 10 μM caused 10% mortality and the most pronounced retardation effect, 5 μM and 2.5 μM resulted in gradual retardation, where the higher concentration was more effective (n = 60, *p <*0.001; Figure 3B). Treatment with 5-CT induced developmental acceleration in all tested concentrations with the maximal effect observed at the third day of incubation (n = 60, *p <*0.001; Figure 3C). Based on this data, the following drugs concentrations were selected for further pharmacological analysis: 2.5 μM of RS67333, 5 μM of 8-OH-DPAT and 1 μM of 5-CT.

Subsequently, we analysed the rate of development, which was expressed as larval size measured after 84 h of incubation with different combinations of 5-HTR agonists and antagonists. Combined application of RS67333 and 8-OH-DPAT induced stronger inhibitory effects on the embryonic development (58 ± 7.4%) than separate applications of each agonist (63 ± 8.9% and 83 ± 5.1% respectively, *p <*0.001; Figure 3D). Developmental retardation caused by RS67333 was partially rescued by co-incubation with 5-CT (72 ± 8.5%, *p <*0.001; Figure 3D).

We also tested whether the developmental effects obtained after application of different 5-HTR agonists were receptor specific. This is particularly important in case of 5-CT and 8-OH-DPAT, because these substances are known to be partial agonists for both 5-HT_7_R and 5-HT_1_R, respectively [56,57]. Developmental retardation induced by RS67333 appeared to be indeed 5-HT_4_R-specific, since it was rescued by the specific receptor antagonist GR113808 (63 ± 8.9% for RS67333 alone vs. to 83 ± 6% for RS67333 + GR113808; Figure 3D). Developmental acceleration induced by 5-CT treatment was not affected by the 5-HT_7_R antagonist SB269970 (Figure 3D), however, it was completely abolished after co-application of highly selective 5-HT_1_R antagonist (S)-WAY100135 (110 ± 4.4% for 5-CT alone vs. 89 ± 5.8% for 5-CT in combination with (S)-WAY100135, *p <*0.001; Figure 3D). This is in line with previous findings demonstrating that 5-CT displays much higher affinity for the 5-HT_1_R than 8-OH-DPAT [24].

The inhibitory effect of 8-ОН-DPAT on the embryonic development was unaltered after co-treatment of larvae with 5-HT_7_R antagonist SB269970 or 5-HT_1_R antagonist (S)-WAY100135. However, combined application of these antagonists resulted in significant reduction of 8-OH-DPAT-mediated developmental retardation to 97 ± 4.3% (Figure 3D). These data suggest that developmental retardation induced by 8-OH-DPAT is most likely mediated via activation of 5-HT_7_R, although its pharmacological properties seems to differ from its mammalian counterpart.

Our pharmacological results demonstrate that activation of different serotonin receptors changed the developmental tempo of freshwater snail *H. trivolvis* in alternative ways. While activation of 5-HT_4_- and 5-HT_7_Rs caused retardation of development, stimulation of 5-HT_1_R resulted in developmental acceleration.

### Developmental effects of 5-HTRs involve cAMP-mediated signalling

The results of pharmacological analysis suggest that 5-HT_1_-, 5-HT_4_- and 5HT_7_Rs are involved in regulation of development in *H. trivolvis* (Figures 2, 3). It has been suggested that invertebrate G protein-coupled receptors might activate similar signalling pathways as their vertebrate homologues. In particular, activation of invertebrate 5-HT_1_Rs results in G_i_ protein-mediated inactivation of adenylyl cyclases (AC) and decrease of cAMP level [58]. Likewise, both vertebrate and invertebrate 5-HT_4_- and 5-HT_7_Rs have been shown to be coupled to stimulatory G_s_ proteins, leading to AC activation and increased cAMP production [59-61].

To test whether cAMP-mediated signalling is involved in regulation of *H. trivolvis* larval development, the specimens were treated with forskolin, a common substance used to increase the intracellular levels of cAMP by activation of AC [62-64]. Application of 10 μM forskolin induced retardation of development to 82 ± 5.8% as compared to the control after 84 h of incubation (n = 100, *p <*0.001; Figure 3E), while the lower concentrations (5 μM and 1 μM) of forskolin had no effect on embryonic development (96 ± 4.3% and 101 ± 3.4% respectively, n = 100; Figure 3E). However, combined treatment of embryos with low concentration of forskolin (1 μM) and RS67333 (5-HT_4_R agonist) resulted in stronger retardation effect of RS67333 compared to the same concentration of RS67333 alone: 36 ± 8% vs. 63 ± 8.9%, (n = 100, *p <*0.001). Moreover, low concentration of forskolin significantly reduced accelerative effect of 5-CT (5-HT_1_R agonist) from 110 ± 4.4% to 99 ± 2.8% (n = 100; Figure 3E).

Incubation of embryos with db-cAMP, cell permeable analogue of cAMP, confirmed the role of cAMP in developmental regulation. After 84 h of incubation, 50 μM db-cAMP induced retardation of development to 89 ± 9.7% as compared to the control (n = 56, *p <*0.001; Figure 3F), while incubation in 10 μM had no effect (98 ± 5.4%, n = 60; Figure 3F). Combined treatment of embryos with 50 μM db-cAMP and RS67333 resulted in stronger retardation effect of RS67333 compared to the same concentration of RS67333 alone: 56 ± 19.5% vs. 63 ± 17.2%, (n = 42, *p <*0.005). Stronger retardation was also obtained after combined application of 50 μM db-cAMP and 8-OH-DPAT compared to the same concentration of 8-OH-DPAT alone: 69 ± 10.4% vs. 78 ± 11.8%, (n = 54, *p <*0.005). Moreover, addition of db-cAMP reduced accelerative effect of 5-CT from 107 ± 3.8% to 99 ± 4.9% (n = 45; Figure 3F).

It is noteworthy that inhibition of both adenylate cyclase and phosphodiesterase by MDL 12330A resulted in 100% death after 24 h (50 μM, n = 68) or 48 h (10 μM, n = 72) of incubation. Low concentration of MDL 12330A (5 μM) resulted in 50% death within three days. Protein kinase A inhibitor H 89 induced 100% death after 24 h of incubation in 10 μM and 20 μM concentrations, while incubation in 1 μM H 89 resulted in non-significant acceleration of development and considerably increased dispersion between length of embryos inside the treated groups (104 ± 12.3%, n = 51, *p* =0.057).

**Figure 4:**
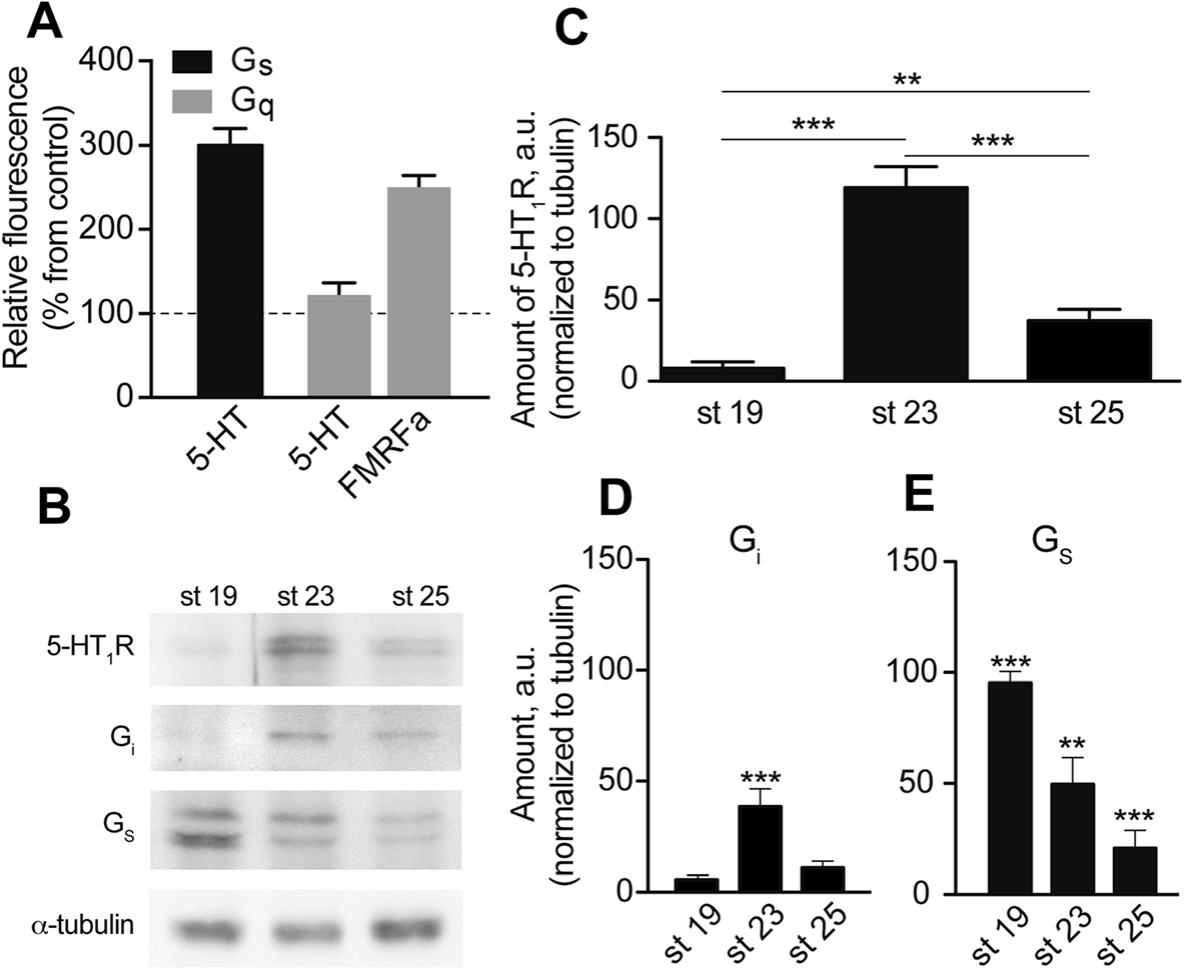
**Expression and activation of G proteins and 5-HT**_**1 **_**receptor during *H. trivolvis *development. (A)** (Eu)GTPγS binding assay demonstrates 5-HT-iduced activation of G_s_, but not G_q_ protein in membrane preparations of *H. trivolvis*. Activation of G_q_ by FMRFamide was used as a positive control. **(B)** Western blot representing proteins from membrane preparations of *H. trivolvis* embryos at different developmental stages. The proteins were visualized with the antibodies against the 5-HT_1_R, G_i_- and G_s_-proteins. **(C-E)** Quantification of protein expression performed by densitometry. Relative amounts of 5-HT_1_R **(C)**, G_i_-protein **(D)**, and G_s_-protein **(E)** are shown at the stages 19, 23, and 25. Expression of 5-HT_1_ receptor and G proteins undergoes changes during *H. trivolvis* development. Maximal expression of 5-HT_1_R and corresponding G_i_-protein was obtained at the beginning of metamorphosis (st 23), while G_s_-protein was maximally expressed at the premetamorphic stage (st 19) and gradually decreases by the metamorphic stage (st 25). Each value represents the mean ± s.d. (n = 4). A statistically significant difference between values is noted (**, *p <*0.01, ***, *p <*0.001).

To verify the role of stimulatory G_s_ protein (a down-stream effector of both 5-HT_4_R and 5-HT_7_R) in developmental retardation we applied the fluorescence-based GTPγS assay performed on the membrane preparations (Figure 4A). Specific coupling of 5-HTR to respective G protein was analyzed by immunoprecipitation (IP) with anti-G_s_ and anti-G_q_ antibodies raised against corresponding G proteins of human origin. 5-HT induced approximately 3-fold increase in (Eu)GTPγS binding to the G_s_-protein and did not induce increase in (Eu)GTPγS binding to the G_q_ protein. Moreover, FMRFamide, a peptide known to stimulate G_q_ protein-linked IP_3_ pathway in fresh-water mollusc [65] induced about 2,5 folds increase in (Eu)GTPγS binding to the G_q_-protein (Figure 4A). Taken together, pharmacological analysis and GTPγS binding assay demonstrate that G protein-mediated cAMP signalling pathway dominates in 5-HTR mediated modulation of developmental tempo in *H. trivolvis* larvae, and the increase of intracellular cAMP level results in developmental retardation.

### Expression patterns of 5-HTRs and related G proteins are different at premetamorphic and metamorphic stages

Pharmacological experiments have demonstrated that the effects of 5-HTRs activation depend on the developmental stage (Figure 2C). One possible explanation for these effects might be the variation in expression patterns of 5-HTRs and/or corresponding G proteins. Therefore, we exploited quantitative real-time PCR to compare the relative expression levels of the mRNA that eventually gives rise to 5HT receptors at different stages of development. Since both 5-HT_1_- and 5-HT_7_Rs have been recently cloned from the genome of *H. trivolvis*[30], it was possible to create specific PCR primers to study their relative expression.

**Figure 5:**
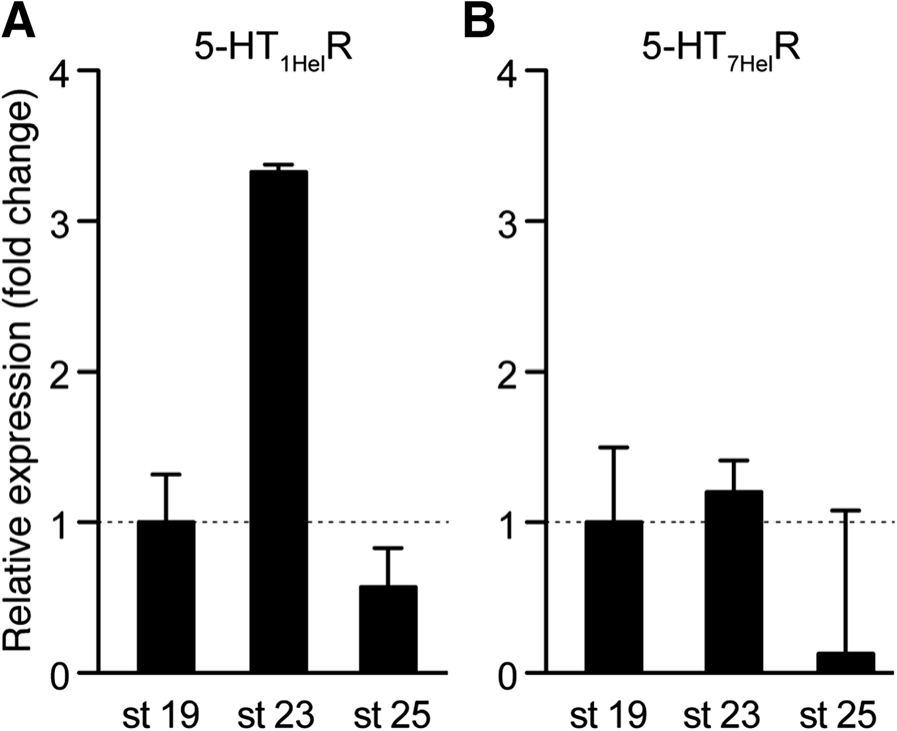
**Expression of the 5-HT**_**1 **_**and 5-HT**_**7 **_**receptors is differently regulated during *H. trivolvis *development.** Relative changes in the amount of mRNA encoding for the 5-HT_1_ and 5-HT_7_ receptors were determined at different developmental stages, including trochophore (st 19), shortly before metamorphosis (st 23) and in the middle of metamorphosis (st 25) using real time quantitative RT-PCR and ΔΔCt method. **(A)** The 5-HT_1_R is transiently unregulated at stage 23 only. **(B)** Relative expression of 5-НТ_7_R gradually down regulated by the metamorphic stage (st 25).

The expression profiles for 5-НТ_1_R and 5-НТ_7_R were analysed at three stages: (*i*) stage 19 long before metamorphosis, (*ii*) stage 23 shortly before metamorphosis and (*iii*) stage 25 that corresponds to the beginning of metamorphosis. The analysis revealed strong transient increase of 5-HT_1_R expressions at the stage 23, while at the earlier and later stages 19 and 25 its expression was significantly lower (up to threefold; Figure 5A). In contrast, expression level of 5-HT_7_R was higher at the premetamorphic stages 19 and 23, and down regulated at metamorphic stage 25 (Figure 5B).

To monitor whether the changes in mRNA level reflect differences in the expression level of corresponding proteins, we performed a quantitative Western blot analysis. Since antibodies against *H. trivolvis* 5-HTRs are not commercially available, we tested antibodies against 5-HT_1A_, 5-HT_4_ and 5-HT_7_Rs of human and murine origin. Application of three different commercially available antibodies against 5-HT_4_- and 5-HT_7_Rs produced no specific signal (data not shown). In contrast, application of the antibody directed against human 5-HT_1A_R resulted in clearly detectable protein band with a molecular weight of around 48 kDa that corresponded to the expected molecular weight of the 5-HT_1_R (Figure 4B). This antibody was therefore used for the quantitative analysis of the 5-HT_1_R expressions at different developmental stages of *H. trivolvis*.

As shown in Figure 4C, the relative amount of the 5-HT_1_R was significantly higher (119 ± 13%) at the stage 23 than at the stage 19 (7.9 ± 4%) and 25 (34.3 ± 7%). Similar expression profile was obtained for the G_i_ protein, the main coupling partner of the 5-HT_1_R (5.7 ± 2% at the stage 19, 38.6 ± 8% at stage 23 and 10.9 ± 3% at the stage 25; Figure 4D). The G_s_ protein that is coupled to both 5-HT_4_- and 5-HT_7_Rs, was strongly expressed during the early premetamorphic developmental stage (stage 19) and gradually down-regulated (up to 5-folds) by the metamorphic stage (95.8 ± 5% at the stage 19, 49.8 ± 10.7% at the stage 23 and 21.2 ± 8% at the stage 25; Figure 4E). These results confirm real-time PCR data (Figure 5B) and strongly suggest that coordinated expression of the receptors and related G proteins might represent a mechanism responsible for the alternative effects of 5-HT at premetamorphic and metamorphic stages of *H. trivolvis* development.

### Activation of 5-HTRs regulates cell proliferation rate of *Helisoma trivolvis* larvae in a stage-dependent manner

**Figure 6:**
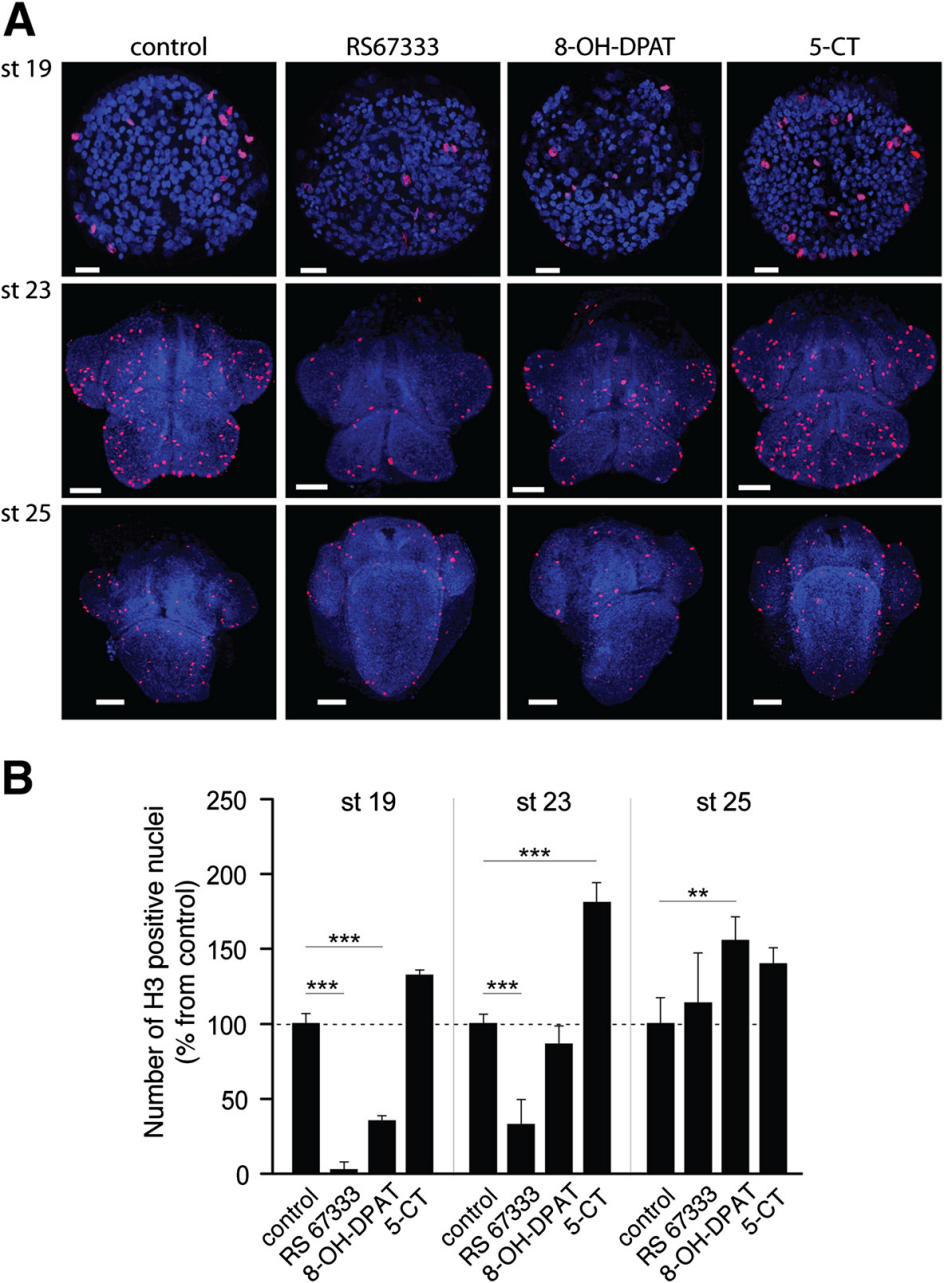
**Effects of different 5-HT receptor agonists on cell proliferation during *H. trivolvis *development. (A)** Representative confocal images of the whole-mount *H. trivolvis* embryos co-stained with DAPI (blue) and the antibody against phospho-H3S28 (red) after incubation with RS67333, 8-OH-DPAT and 5-CT (5 μM each). Staining with anti-phospho-H3S28 antibody allows visualization of dividing cells. Scale bars are 20 μM for the stage 19, and 50 μM for the stages 23 and 25. **(B)** The relative number of dividing cells at stages 19, 23 and 25 after 10 h of incubation with RS67333, 8-OH-DPAT and 5-CT. Each value represents the mean ± s.e.m. (n = 10). A statistically significant difference between values is noted (** *p <*0.01, *** *p <*0.001).

The above analysis of 5-HTRs and corresponding G proteins indicates that cAMP is critically involved in regulation of the developmental tempo. Cyclic AMP is known to be one of the regulators of cell division rates [66-71] suggesting that agonist induced changes in the *H. trivolvis* developmental rate might correspond to the changes in cell division rates. To test it, we studied the effects of different 5-HTR agonists on the number of dividing cells at the developmental stages 19, 23 and 25. Incubation with different agonists was followed by the staining with the antibody against the phosphorylated form of H3 histone, which binds to the histone molecules only at the prophase and metaphase stages of mitosis [72-74], and thus allows visualization of dividing cells (Figure 6A).

Treatment of animals with 5-HT_4_R agonist RS67333 (5 μM) at premetamorphic stages 19 and 23 significantly inhibited cell divisions to 2.5 ± 5.6% and 33 ± 16.4% respectively (Figure 6A, B, n = 10, *p <*0.0001 and *p <*0.001 respectively). Treatment at the metamorphic stage 25 had no significant influence on the number of dividing cells (114 ± 33.1%, n = 10; *p* >0.05; Figure 6A, B). Incubation of animals with 5-HT_7_R agonist 8-OH-DPAT (5 μM) at the stage 19 resulted in significant reduction in the number of mitotic cells to 35 ± 12.3% (Figure 6A, B, n = 10; *p <*0.001). However, this inhibitory effect was weaker at the stage 23 (86 ± 36.5%, n = 10; *p* >0.05) and even reversed to induce stimulation of cell divisions at the stage 25 (156 ± 49.2%, n = 11; *p <*0.01; Figure 6). On the contrary, incubation of embryos with 5-HT_1_R agonist 5-CT resulted in a strong increase in the number of mitotic cells at stage 23 (133 ± 35.2% at the stage 19, n = 10, *p* >0.05; 181 ± 40.5% at the stage 23, n = 10, *p <*0.001 and 139 ± 36.2% at the stage 25, n = 11, *p* >0.05; Figure 6A, B) when expression of 5-HT_1_R and G_i_ protein reached the maximum values (Figures 5A, 4C, D). Thus, at premetamorphic stages, the retarding effects of RS67333 and 8-OH-DPAT are at least partly based on receptor-mediated suppression of cell divisions, and the accelerating effect of 5-CT is based on receptor-mediated increase of cell divisions.

## Discussion

### Development of *H. trivolvis* larvae includes transition from premetamorphic to metamorphic stages

Freshwater gastropod snails develop from the first cleavage to the juvenile stage entirely within egg capsules. Thus, unlike the majority of other mollusks, *H. trivolvis* does not have free-swimming larval stages [75,76]. However, sufficient vestiges of larval phases and similarities in timing of the appearances of various other structures exist such that parallels with developmental processes in marine gastropods can nonetheless still be inferred [77]. It has been demonstrated that *H. trivolvis* possesses specialized larval characters such as the apical 5-HT-immunoreactive neurons belonging to the ASO [28,78,79]. We demonstrate that these ASO neurons as well as dorsolateral and pedal ciliary bands are transient structures, suggesting that they are homologous to the respective structures of molluscan free-swimming larvae [1,80,81]. It has been postulated that all metamorphoses share four main components: (1) the differentiation of juvenile/adult structures, (2) the degeneration of larval structures, (3) metamorphic competence, and (4) change in habitat [82]. Taken together, our morphological and behavioural data demonstrate that encapsulated larvae of *H. trivolvis* possess all these components and undergo the transition from the premetamorphic to metamorphic stage.

### 5-HT_1_-, 5-HT_4_- and 5-HT_7_-like receptors are involved in regulation of *Helisoma trivolvis* development

Several previous reports were focused on the effects of various neurotransmitters, such as 5-HT, GABA, acetylcholine, dopamine and histamine on metamorphosis of aquatic invertebrates [24,83-89]. It has been shown that in a broad range of species all these transmitters can modulate the processes of metamorphosis and settlement of competent larvae. Thus, 5-HT was shown to trigger metamorphosis and induce settlement in hydroids, ascidians, barnacles and molluscs [21-26,30].

5-HT was detected in early larvae of molluscs long before the onset of metamorphosis [2,41,42,44,77,78,90]. We have shown that endogenous 5-HT produced by the apical sensory neurons of larval *H. trivolvis* can either retard or accelerate the developmental tempo (the speed of transition from one stage to another) depending on the stage the larva passes [35,36]. Our data suggest that internal 5-HTRs are present from the early stages of larval development, and probably different sets of 5-HTRs are active at different developmental stages. Two receptors belonging to the 5-HT family, 5-НТ_1_R and 5-НТ_7_R, have been cloned for *H. trivolvis*[30], and involvement of both receptors in cilioexcitatory response to serotonin was suggested [29]. However, no experimental data about their functional role was yet provided.

In the present study, we utilized combination of pharmacological, biochemical and molecular approaches for characterisation of *H. trivolvis* 5-HTRs involved in 5-HT-mediated changes in developmental tempo. The effects of commonly used agonists and antagonists for 5-HT_1_-, 5-HT_4_- and 5-HT_7_Rs obtained in our experiments strongly suggest that receptors of these subfamilies are functionally active in *H. trivolvis* larvae. It is supported by the following observations: (*i*) All used agonists demonstrated their functional effects in low (μM) concentrations and in dose-dependent manner. (*ii*) If applied simultaneously, different agonists demonstrated expected summation of their effects: RS67333 and 8-OH-DPAT showed additive action, and RS67333 and 5-CT decreased the effects of each other. (*iii*) Agonist-mediated effects were attenuated by the application of corresponding antagonists.

It is possible that changes in the developmental tempo observed in experimental animals may result from heterochrony rather than developmental retardation or acceleration. However, we are quite confident in that heterochrony can be neglected by several reasons. First, we always checked the dispersion in developmental stages between individual animals within experimental and control groups by inspecting the set of morphological, morphometric and behavioural characters. Second, we always measured the dispersion in the length of embryos. One of characteristic features of *H. trivolvis* that makes this animal a suitable experimental model is highly synchronous embryonic development with low divergence between individual embryos within one egg mass (see Table 1), and this divergence in experimental groups never significantly differed from the controls. Moreover, any disproportionality in growth and malformations were never observed in experimental animals.

PCR and Western Blot analysis confirmed the presence of 5-НТ_1_ and 5-НТ_7_ receptors at all examined developmental stages. The presence of 5-HT_4_ like receptor was suggested based on the response to specific drugs. Although invertebrate receptors can have different affinity for drugs if compared to the vertebrate ones, our data suggest that *H. trivolvis* receptor indeed belongs to the 5-HT_4_ subfamily. First, action of the corresponding agonist RS67333 was strictly concentration-dependent; it was rescued by the specific antagonist (GR113808) and was not rescued by other antagonists. Second, this receptor is coupled to the cAMP signalling pathway since the effect of RS67333 significantly increased after co-application with db-cAMP and forskolin though low concentrations of db-cAMP and forskolin alone were non-effective.

Pharmacological analysis shows that inhibitory action on developmental tempo of pharmacological agents that mimic the effects of endogenous 5-HT is mediated via activation of 5-НТ_7_- and 5-HT_4_Rs, whereas accelerating action is mediated through the activation of 5-НТ_1_R. It is known that 5-HT or some 5-HTRs agonists stimulate rotation of *H. trivolvis* embryos mixing the egg capsule fluid, thereby enhancing delivery of environmental oxygen to the embryo [27,29]. It might be suggested that changes in developmental tempo can be caused by increase or decrease in embryo rotation and thereby changes in larval metabolism. However, our previous work demonstrated that external 5-HT strongly stimulated embryonic rotations, while it did not influence the developmental rate [35]. Thus, only endogenous 5-HT produced by the apical neurons in response to the external stimulus induces the changes in developmental tempo, whereas exogenous 5-HT acts on the surface ciliary structures and stimulates rotations.

Our data demonstrate that the cAMP pathway is involved in regulation of developmental tempo, since elevation of the cAMP level by forskolin and application of db-cAMP resulted in developmental retardation. Co-application of forskolin and db-cAMP enhanced the effects of 5-HT receptor agonists that are positively coupled to AC. Besides, our coupling assay, demonstrated 5-HT-mediated activation of G_s_ (cAPM pathway) but not G_q_ (IP_3_ pathway) protein. To the contrary, artificially raising the intracellular Ca^2+^ concentration caused a 5-HT-like increase in the cilia-driven rotational movements, while treatments that elevate the concentration of intracellular cAMP, did not mimic the 5-HT-induced increase in the ciliary beat frequency [91]. Thus, the two processes, stimulation of ciliary locomotion and modulation of developmental tempo possibly utilise different physiological mechanisms.

### 5-HTRs modulate the cAMP-dependent regulation of cell divisions in *Helisoma* larvae

5-HT_1_-, 5-HT_4_- and 5-HT_7_Rs differ in their intracellular signalling in that 5-HT_1_R inhibits the activity of AC via activation of inhibitory G_i_ proteins, while 5-HT_4_- and 5-HT_7_Rs stimulate AC via activation of stimulatory G_s_ protein [92,93]. We demonstrate that both G_i_ and G_s_ proteins are present in *H. trivolvis* larvae and cAMP is involved in signal transduction pathway of 5-HT. This is directly confirmed by serotonin-induced (Eu)GTPγS binding to the G_s_-protein as well as by pharmacological analysis.

Importance of cAMP in development is well known. In the amoeba *Dictyostelium discoideum*, production of сAMP induced the transition from growth to development [94]. Elevation of cAMP in crayfish postponed molting and prolonged current life stages [95]. Increase of cAMP production by an external signal (natural or artificial) induced metamorphosis in competent larvae of gastropod mollusc *Haliotis rufescens*[84]. cAMP pathway mediates settlement of larvae in marine annelids [63,64]. Metamorphosis is mediated by a combination of both AC/cAMP and PI/DAG/PKC pathways in larvae of the barnacle *B. amphitrite*[96-98]. Our observation that modulation of cAMP level through activation of 5-HT receptors influences the tempo of larval development in *H. trivolvis* is in agreement with the above listed data. How changes in the cAMP level can modulate the complex process of larval development, including cell divisions, growth and differentiation of tissues and organs? Our data demonstrate that at least the rate of cell divisions in *H. trivolvis* is under control of 5-HTRs. Activation of receptors which rise the cAMP level (5-HT_4_- and 5-HT_7_Rs) resulted in reduced number of dividing cells, whereas activation of 5-HT_1_R, which decreased the cAMP level, increased the number of cell divisions. These results are in accordance with the data demonstrating that cAMP can regulate mitosis in various cell types by inhibition or even arrest of cell cycle progression [66-71]. Noteworthy, the reduction in cell divisions observed at premetamorphic stages correlates with the higher expression level of G_s_ protein-coupled 5-НТ_7_R. Also the pronounced increase of cell divisions obtained shortly before metamorphosis match well with the expression peak of 5-НТ_1_R. Taken together these results suggest that cAMP might have a conservative function in regulation of cell cycle among broad variety of species.

### Coordinated expression of 5-HTRs and coupled G proteins can regulate transition of trochophore larvae from precompetent to competent stage

Larvae that are able to undergo metamorphosis in response to specific cues are called “competent”. Metamorphic competence was defined as “the developmental potential of a larva to undergo the radical transition from a larval to a juvenile/adult body plan in response to internal or external signals” [83]. Noteworthy, in one species, the duration of premetamorphic (precompetent) stage may vary from several days to several weeks [17,99], and even from weeks to more than a year [100]. The question about basic differences between precompetent and competent larvae has been debated for a long time [16,17,101]. In the majority of aquatic animals, no specialized morphological structures, which are absolutely necessary for metamorphosis, appear in competent larvae. Only in few species among investigated, transition to larval competence is accompanied by appearance of specific morphological characters (i.e. propodium and red spots on the mantle of *Aplysia*) [16]. More often, no morphological correlates can be found, and the larva becomes competent when it starts to react to external signals that induce metamorphosis. The ASO and its serotonergic neurons have been experimentally proven to be required for detection of these signals and settlement initiation [49]. However, in all species investigated so far, the ASO appears long before metamorphosis and the larva does not react to the signals until it becomes competent [16,25,39]. Several recent studies have employed genomic, transcriptomic and proteomic approaches to elucidate mechanisms underlying the metamorphic competence and settlement. Although these studies have produced a large number of candidate mechanisms [16], they have not been confirmed by functional analysis.

**Figure 7:**
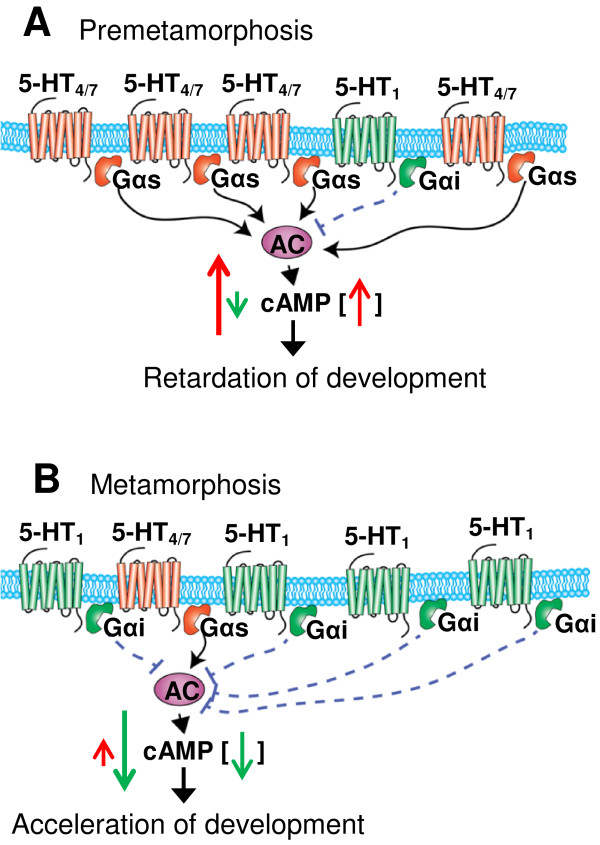
**Proposed mechanism of stage dependent 5-HT receptor mediated regulation of larval development in *H. trivolvis. *(A)** At premetamorphic stage, G_s_-coupled serotonin receptors (such as 5-HT_4_R and 5-HT_7_R) and their down-stream effectors are highly expressed and their relative amount exceeds that of 5-HT_1_ receptor and coupled G_i_-protein. Thus, the ligand activation of different 5-HT receptor subtypes results in overall elevation of cAMP level (red arrow in square brackets), which leads to decreased rate of cell divisions and finally results in developmental retardation. **(B)** At metamorphic stage, 5-HT_4_R, 5-HT_7_R, and coupled G_s_-protein become down-regulated, while expression of 5-HT_1_R and G_i_-protein increases. Relative amount of 5-HT_1_R now exceeds that of 5-HT_4_R and 5-HT_7_R, and ligand stimulation results in overall reduction of cAMP level (green arrow in square brackets), and subsequent developmental acceleration. According to the proposed mechanism, endogenous serotonin released from the ASO neurons in respond to external cues induces opposite effects at premetamorphic and metamorphic stages.

Experimental data presented in our study suggest that transition from a latent, noncompetent state to an alert competent state (when the larva is ready to respond to metamorphic signals) can be provoked by changes in the expression level of particular serotonin receptors and respective G proteins. As a result of such regulated expression, the external signal that previously retarded the development or was meaningless starts to facilitate the development or triggers metamorphic events. Both processes can be initiated by activation of the same apical sensory neurons that release 5-HT. Based on our results, we propose a novel mechanism by which serotonergic neuroendocrine signalling can differentially regulate development of *H. trivolvis* at precompetent and competent stages (Figure 7).

According to the proposed mechanism, premetamorphic and metamorphic larvae differ in the expression pattern of 5-HTRs and G proteins. At premetamorphic (precompetent) stages, expression of 5-HT_4_- and 5-HT_7_Rs and corresponding G_s_ protein prevails while expression of 5-HT_1_Rs and corresponding G_i_ protein is low. In the course of development, 5-HT_1_Rs and G_i_ proteins undergo up-regulation and become preferential at metamorphic (competent) stage. Depending on what types of receptors (5-HT_4_- and 5-HT_7_Rs or 5-HT_1_Rs) and their downstream partners (G_s_- or G_i_-proteins) prevail at a particular developmental stage (premetamorphic or metamorphic), endogenous 5-HT released from serotonergic neurons of the ASO in response to external cues will either result in the increase of cAMP level and retardation via modulation of cell cycle progression at premetamorphic stage, or result in decrease of cAMP level and acceleration of the developmental tempo at metamorphic stage.

Besides serotonergic neurons, cells producing dopamine [54], histamine [89], as well as neurons containing nitric oxide synthase [102,103] and peptides of the RFamide family [104-106] were found in the nervous system of invertebrate larvae, particularly in the ASO, and some of these neurotransmitter systems were shown to act in coordination during settlement and metamorphosis of free swimming invertebrate larvae [82,89,107]. It was also shown that effectiveness of 5-HT and acetylcholine in induction of metamorphosis in larvae of bivalve mollusc *Ruditapes phillipinarum* was low in precompetent pediveligers and significantly increased in competent pediveligers [25]. Besides, the effects of putative dopamine antagonists on metamorphosis in the marine gastropod *Crepidula fornicata* changed dramatically as larvae aged [87]. The authors suggested that a special sensory system develop in competent pediveligers to recognize specific cues for settlement and metamorphosis [25]. However, they left the question about physiological and molecular mechanisms underlying the observed phenomena open. We speculate that stage-dependent effects of neuroactive substances at pre- and metamorphic stages uncover a molecular mechanism underlying transition to metamorphic competence in aquatic invertebrates with biphasic life cycle. The larva becomes competent when coordinated changes in expression patterns of specific neurotransmitter receptors and their downstream partners take place in the course of development, so the larva becomes sensitive to metamorphic cues. Our results together with the above listed data suggest that 5-HT, dopamine, acetylcholine and other neurotransmitter systems might be involved in this regulation, and these systems might act independently or in coordination. The multiplicity and coordinated action of neurotransmitters and neurotransmitter receptors controlling developmental transitions, settlement and metamorphosis in aquatic invertebrates are still the subject of speculation and require further investigation.

## Conclusions

Our study describes the changes in 5-HT receptors and corresponding G proteins in the course of development of freshwater pulmonate snail *H. trivolvis*. We demonstrate that larval development of *H. trivolvis* includes transition from premetamorphic to metamorphic stages, and share the main features of metamorphosis with free swimming aquatic larvae. We revealed three types of 5-HT receptors (5-HT_1_-, 5-HT_4_- and 5-HT_7_-like) to be expressed and functionally active at premetamorphic (trochophore and veliger) and metamorphic (veliconcha) stages. Stimulation of these receptors modulated cAMP-dependent regulation of cell divisions. Expression patterns of 5-HT receptors and respective G proteins underwent coordinated changes during development. Thus, expression of 5-HT_4_- and 5-HT_7_-like receptors and their downstream G_s_ protein was down-regulated during the transition of pre- to metamorphic stage, while expression of 5-HT_1_ -like receptor and its downstream G_i_ protein was upregulated. In accordance with relative amount of these receptors at different developmental stages, stimulation of 5-HTRs at premetamorphic stages caused developmental retardation, while their stimulation at metamorphic stages caused developmental acceleration. The proposed model suggests a novel molecular mechanism that underlies transition of the larva from premetamorphic (noncompetent) to metamorphic (competent) state.

## Abbreviations

5-CT: 5-carboxyamido-tryptamine maleate; 5-HT: 5-hydroxytryptamine, serotonin; 8-OH-DPAT: 8-Hydroxy-2-dipropylaminotetralin hydrobromide; AC: Adenelyl cyclase; ASO: Apical sensory organ; cAMP: Cyclic adenosine monophosphate; DOI: 2,5-dimethoxy-4-iodoamphetamine; n.e.d.: Normal embryonic development; PB: Phosphate buffer.

## Competing interests

The authors declare that they have no competing interests.

## Authors’ contributions

EGP, EEV and KG conceived the project and designed experiments; EEV, MYuK performed pharmacological experiments; KG and EEV performed biochemical and molecular biological experiments; EI made the cell division analysis. EEV and LPN performed microscopy and morphological analysis. EEV, KG, EGP and LPN wrote the manuscript. All authors contributed to the manuscript preparation and have read and approved its final version.

## Supplementary Material

Additional file 1 **Episode of gliding locomotion of the control *H.trivolvis embryo.*** At the developmental stage 26 (middle metamorphic stage), the embryo attaches the foot to the inner surface of the egg capsule and creeps along it. Heart beatings, muscles contractions, and mixing of capsular fluid by surface cilia are visible. Click here for file

Additional file 2 **Episode of rotational swimming of the control *H.trivolvis *embryo.** At the developmental stage 26 (middle metamorphic stage), the embryo sometimes detaches from the inner surface of the egg capsule and switches to rotational swimming. Click here for file

Additional file 3 **Episode of rotational swimming of the *H.trivolvis *embryo after treatment with RS67333.** The treatment started at the developmental stage 19 and the embryo was recorded after 84 hours. The embryo is at the stage 23 (premetamorphic veliger) and differs from the controls in smaller size, different shape, and lack of pigmented eyes. No heart beatings and muscles contractions can be seen. Click here for file

Additional file 4 **Episode of gliding locomotion of the *H.trivolvis *embryo after treatment with 5-CT.** The treatment started at the developmental stage 19, and the embryo was recorded after 84 hours. The embryo is already at the stage 27 (the end of metamorphosis). It creeps along the inner surface of the egg capsule using the fully developed foot. The shell covers the whole visceral mass. Heart beatings, pneumostome openings and pigmented eyes at the base of the tentacles are seen. Click here for file
